# Traceable Security-by-Design Decisions for Cyber-Physical Systems (CPSs) by Means of Function-Based Diagrams and Security Libraries

**DOI:** 10.3390/s23125547

**Published:** 2023-06-13

**Authors:** Sarah Fluchs, Emre Taştan, Tobias Trumpf, Alexander Horch, Rainer Drath, Alexander Fay

**Affiliations:** 1 Institute of Automation, Helmut-Schmidt-University, 22043 Hamburg, Germany; alexander.fay@hsu-hh.de; 2 admeritia GmbH, 40764 Langenfeld (Rheinland), Germany; 3 Faculty of Technology, Pforzheim University, 75175 Pforzheim, Germany; emre.tastan@hs-pforzheim.de (E.T.); rainer.drath@hs-pforzheim.de (R.D.); 4 HIMA Paul Hildebrandt GmbH, 68782 Brühl, Germany; t.trumpf@hima.com (T.T.); a.horch@hima.com (A.H.)

**Keywords:** cyber-physical systems, industrial control systems, cybersecurity, security by design, visualization, function-based engineering

## Abstract

“Security by design” is the term for shifting cybersecurity considerations from a system’s end users to its engineers. To reduce the end users’ workload for addressing security during the systems operation phase, security decisions need to be made during engineering, and in a way that is traceable for third parties. However, engineers of cyber-physical systems (CPSs) or, more specifically, industrial control systems (ICSs) typically neither have the security expertise nor time for security engineering. The security-by-design decisions method presented in this work aims to enable them to identify, make, and substantiate security decisions autonomously. Core features of the method are a set of function-based diagrams as well as libraries of typical functions and their security parameters. The method, implemented as a software demonstrator, is validated in a case study with the specialist for safety-related automation solutions HIMA, and the results show that the method enables engineers to identify and make security decisions they may not have made (consciously) otherwise, and quickly and with little security expertise. The method is also well suited to make security-decision-making knowledge available to less experienced engineers. This means that with the security-by-design decisions method, more people can contribute to a CPS’s security by design in less time.

## 1. Introduction

All too often, IT security is left as an end users’ problem. It is commonly accepted that all products and systems either already have security flaws or, if none are known yet, security flaws will be discovered within the products’ lifespans, and that it is the end users’ responsibility to operate the system securely. This has recently been called out as a major problem by the USA’s Cybersecurity and Infrastructure Security Agency (CISA) [1].

The term for shifting cybersecurity considerations from a system’s end users to its engineers is “security by design”. Security by design means that IT/OT security issues are identified and solved during the design phase of the system, rather than as an afterthought when the system is already operational. Policy makers in many countries have recently included security by design more prominently in strategy papers and regulation. For example, it is the main pillar in the European Union’s draft for the Cybersecurity Resilience Act [2] as well as in the US National Cybersecurity Strategy [3]. In April of 2023, national security authorities from the USA, Canada, the Netherlands, Great Britain, Australia, New Zealand, and Germany published a document stating the principles of security by design and default, expressing a common interest in and priority for the topic [4].

Like for information technology and software, the importance of security for cyber-physical systems (CPSs) is on the rise: Due to the more intensive networking of CPSs with increasing use of internet technologies and common IT products, the possible and easily accessible communication interfaces to CPSs are increasing. This makes them more vulnerable and worthwhile for criminal cyber-attacks and increases the importance of effective security measures to prevent such attacks.

However, security by design for CPSs and, more specifically, for industrial control systems (ICSs) differs from security by design for software. Like for CPS and ICS, the system’s design cannot be carried out solely at the component’s manufacturer. A large part of the engineering process is carried out when multiple components are integrated into a more complex system for automating a production process [5,6] such as producing a pharmaceutical or an infrastructure service such as wastewater treatment or power distribution. Therefore, if the responsibility for a system’s security is to be shifted away from the end user for CPSs and ICSs, engineers working at components manufacturers, integrators, and plant owners need to be empowered to perform security by design.

However, these engineers currently do not have the obligation to embed security, and consequently they neither have the time, the security expertise, nor the processes or methods to consider security timely in their design [7,8,9]. Therefore, empowering engineers for security by design must include giving them guidance, providing know-how, and saving them time. Empowering engineers to consider security during design not only increases the products’ security; it also reduces cost: each security vulnerability that is found after the design is finished causes a significant workload in eliminating the problem, communicating with product users, and limiting reputational damage—all unplanned and under time pressure.

The overarching goal of security by design is to decide which security measures are needed (and, usually the harder decision: which are NOT needed) to create a defensible system [10]. The required guidance for security by design can be divided into four challenges around these central decisions:

1. **Decision identification:** The challenge is to identify which of the many system characteristics and design decisions affect the system’s security.
2. **Decision making:** The challenge is to have all relevant information handy to make profound, informed, and systematic security decisions.
3. **Decision tracing:** The challenge is to clearly understand and document the decisions’ rationales.
4. **Decision timing:** The challenge is to make the security decisions regarding a certain design aspect early enough, i.e., when this design aspect can still be influenced. This means that the security decision making needs to be integrated early enough into the existing engineering workflow.

This paper addresses all but the last of these four challenges, because decision timing methods are still being validated by the authors at the time of this writing. Therefore, the research questions for this paper are: *How can CPS/ICS engineers with limited time and security expertise be guided to (a) identify security decisions during their engineering workflows (**decision identification**), (b) make security decisions autonomously (**decision making),** and (c) make security decisions that can be reproduced and revised later and/or by others (**decision tracing**)?*

It should be noted that the research questions do not address the quality of the security decisions, but only the engineers’ ability to identify and make informed security decisions autonomously, early enough in the design process, and document their rationales. While the quality of the resulting security decisions certainly matters, it is very difficult to measure objectively. In addition, the evaluation of whether the security decisions are implemented correctly is out of scope of this article.

The remainder of this paper is organized as follows: In Section 2, the state of the art for security decision identification, making, and tracing is summarized. Section 3 contains the proposed concept to guide engineers’ security-by-design decisions, built around the cornerstones of new, function-based security diagrams and security parameters. In Section 4 and Section 5, the results of the concept validation at HIMA, a specialist for safety-related automation solutions, are presented and discussed. The validation is carried out as a case study with a mix of quantitative and qualitative metrics to measure the concept’s effectiveness in addressing the research questions. Section 6 concludes the paper by pointing out the authors’ contribution to the problem introduced earlier and outlines future research.

## 2. State of the Art

Before the state of the art of security decision identification, making, and tracing can be discussed, it must be made clear which kind of security decisions are in focus. There are two kinds of security decisions: Security design decisions (in scope of this paper) and security operations decisions (not in scope). Security operations decisions are decisions that affect the security of a system in its operation phase, such as monitoring for ongoing attacks and newly disclosed vulnerabilities and deciding what to do about them. The goal of security operations decisions can be summarized as “defend the system”, while the goal of security design decisions can be summarized as “build a defensible system”.

A significant imbalance becomes apparent when the maturity of methods and tools to support security operations decisions is compared to those to support security design decisions. For security operations decisions such as “do we need to patch this vulnerability” or “how do we react to a suspicious change in data traffic”, there are countless examples for supporting tools: security incident and event monitoring (SIEM), intrusion detection and prevention (IDS/IPS), vulnerability scanners, antivirus solutions, etc.

For security design decisions, methods and tools are less mature. In the following, the state of the art is summarized separately for (1) decision identification and (2) decision making and tracing.

### 2.1. Decision Identification

For identifying security design decisions for CPSs, i.e., identifying which security measures to decide about, the state of the art is using security requirements checklists.

Often, these checklists are provided as standards or technical reports. Some of the most popular lists are multiple parts of the ISA/IEC 62443 standard series (part 2-1 [11] for asset owners, parts-4-1 [12] and -4-2 [13] for components, and part-3-3 [14] for integrated systems), ISO/IEC 27001:2022 [15], or the NIST Cybersecurity Framework [16].

More specific checklists exist, for example, in [8], where Eckhart et al. list a mix of security processes and technologies to be considered during CPS engineering. Even Ross Anderson’s book “Security Engineering” [17] draws much of its strength from providing checklists for security measures that should be considered, along with contexts and anecdotes to help the reader understand why they are needed.

The checklist approach for identifying security decisions has a long history outside of CPSs. In a paper published in 1993, Baskerville analyzed security design methods for information systems and categorized them in three generations [18]: checklist methods are the first generation, first used in the 1970s.

Checklists are a simple, effective approach for making sure important decisions are not overlooked. Obviously, this only works if all relevant decisions are on the list, causing lists to grow longer. Only rarely are requirements removed from these checklists, as Dale Peterson pointed out in his keynote at the S4x23 ICS security conference [19].

Nevertheless, security requirements checklists are incomplete because they tend to only cover technologies and best practices that are explicitly in the security realm—authentication, encryption, etc. However, there are additional, “undercover” security decisions that are not commonly perceived as security-relevant but can have security impacts anyway. This may be low-level decisions such as whether the design of a safety shutdown is mechanical or electronical—the latter being more prone to being hacked remotely. Another example is the question of how to determine if a sensor value can be trusted and recover states from corrupted measurements [20]. This is security-relevant because state-of-the-art technologies for validating sensor values may not be capable of detecting malicious manipulations if a sensor is being attacked. In addition, a large source for suchlike security decisions are “insecure-by-design” features [21]—functional features that are implemented purposefully to meet functional requirements but can also be exploited in a security attack, such as the decision to enable the update of PLC logic during operations.

The fact that suchlike “undercover” security decisions are not systematically identified nowadays means that engineers are often not aware that what they perceive as a functional decision is a security decision as well. Thus, these security decisions are made, but without security in mind.

To make it worse, “undercover” security decisions are typically made very early in the design process, making them difficult to revise if their security relevance is caught late in the engineering workflow [7].

### 2.2. Decision Making

Compared to identifying security decisions, making and tracing them are more complex, and the state of the art covers a broader spectrum of approaches. As discussed earlier, checklists are reasonable for identifying security decisions, but when it comes to making decisions, they provide little guidance. The only way a checklist can be used to guide decision making is by assuming all items on the list need to be implemented, so every identified decision defaults to a “yes”. As Baskerville pointed out in [18], when checklists were first used for security decision making, this was acceptable, because the number of available security solutions was very limited, so applying all of them was actually realistic. Even in 1993, Baskerville stated that with the increase in available security solutions, such an approach was no longer realistic. Now, 30 years later, saying “yes” to all theoretically available security solutions is even less realistic. Instead, security decision making—picking the solutions that have the greatest benefit—has become more relevant.

In Ref. [10], the authors described four different categories of security decision making: compliance-driven, risk-driven, goal-driven, or library-supported. Which of the four decision-making paths apply is each organization’s individual, strategic choice. Risk-driven and goal-driven decision making involves defining one’s own security priorities, expressed by a risk or unwanted event to prevent, or a design goal to meet. Compliance-driven security decisions are more other-directed and, in case legally binding regulations must be complied with, non-negotiable. Relying on library-supported decisions makes sense if an organization’s systems are so standardized (and/or the decision-making process is so mature) that it pays off to standardize security decisions too. The state of the art for each decision-making path is presented in the remainder of this section. An overview of advantages and limitations of each approach is given in Table 1.

**Compliance-driven security decision making** essentially means checklist-based decision making, with the only addition that the checklist is part of a law or regulation that needs to be complied with. Like checklist-based approaches, it does not provide much guidance for security decision making, but in the context of an increasing amount of regulation for critical infrastructure security (NIS 2 [22] and RCE [23] directives in the European Union, PPD-21 [24] directive in the US, and equivalents in many other countries), compliance-driven decision making is one of the most common ways CPS security decisions are made in practice and therefore needs to be mentioned. Examples for checklists being followed in today’s compliance-driven security decision making for CPSs are given in Section 2.1.

**Table 1:** Comparison of decision-making categories.

Category and Example References	Advantages/Use Cases	Limitations
**Compliance-driven security decision making** based on requirements lists such as - Parts of the IEC 62443 series [11,12,13,14] - ISO/IEC 27001 [15] - NIST Cybersecurity Framework [16] - Best practices as in [17] - Secure development practices as in [8,12]	- Efficient if there are clear, maybe even legally binding, security requirements catalogs. - Not much security expertise required.	- Not much guidance for decision making. - Decision making is only as good as the consulted regulation/catalog. - Decision is not traceable beyond referencing the applicable regulation and requirement.
**Risk-driven security decision making** Abundance of publications, surveys, e.g., in - Cherdantseva et al. [25] - Lemaire et al. [26] - Fluchs and Rudolph [27,28]	- Flexible if an organization wants to set its own security priorities based on events that must not occur or risks to be mitigated. - Good for management communication. Fits in well with corporate risk management that may be used in other areas (e.g., finance).	- Relies on system understanding, which is often neglected or oversimplified (mechanistic world view) in existing methods. - Existing methods are fragmented; there is no comprehensive method that builds upon the system model all the way through to decision making. - Improvements (more holistic system models, more systematic decision making based on these models) all significantly increase the amount of information to be created and digested for decision making and tracing. Thus, tool support is needed to keep decision making efficient.
**Goal-driven security decision making** - In software requirements engineering [29,30,31,32,33] - In model-based systems engineering [34,35] - Tropos/i* [36,37] - use case-based approach [24,38,39,40]	- Flexible if an organization wants to set its own security priorities based on security goals or features that the systems should have. - Intuitive for engineers, especially if they are used to (goal-based) requirements engineering.	- More complex system models including human stakeholders and their intentions are needed. - CPS engineers are often not familiar with goal-based design.
**Library-supported security decision making** - Patterns [9,41,42,43] - Common Criteria protection profiles [44] - Ontologies [45,46,47] - MITRE ATT&CK for ICS [48]	- Efficient if an organization has standardized systems and/or very mature security decision-making processes so that security decisions can be standardized. - Encapsulates security knowledge and makes it re-usable, so security experts do not need to be consulted for every project.	- Library-supported decisions and their traceability is only as good as the library. - Libraries need to be maintained and kept up-to-date. - Organizing knowledge in libraries so that security decision makers actually find what they need with acceptable effort is not trivial and most likely not doable without tool support.

**Risk-driven security decision making** has historically been the first reaction to the limitations of compliance-driven security decision making for software security [18]. Baskerville draws the following timeline for information systems security: In the 1970s, risk assessments were introduced as an addition to checklist-based decision-making approaches as a means of finding the measures on the checklist with the best cost–benefit ratio. However, in the 1980s, these methods evolved into more sophisticated risk-based approaches. These added two additional steps in the workflow before arriving at security requirements: First, describing a system by dividing it into “assets”; second, identifying threats to these assets. Security requirements do not come in before the first step, where they are introduced to mitigate the identified threats. At that point, checklists are still being used as a source of inspiration [18].

Most of the publications around CPS security decision making still follow this scheme. There is an abundance of publications around risk-driven security decision making. For the sake of brevity, some overview studies are listed below, along with their main findings, especially those regarding weaknesses of the existing risk-driven approaches.

The first weakness was already described by Baskerville in 1993: He calls this way of decision making “mechanistic”, because the assets are identified based on a mechanistic system view, dividing a system into its physical components and disregarding everything else. This, he wrote, causes two problems: First, requiring a mechanistic system model implies that the physical system must be fully specified when the security design is being carried out, causing it to be (too) late in the engineering workflow and completely isolated from the functional system design. In addition, modeling and understanding the entire physical system in interconnected assets are complex, time-consuming, and require a well-trained “super analyst” capable of overlooking the entire system specification in detail [18].

Turning more specifically to CPSs, Cherdantseva et al. reviewed existing CPS risk assessment methods in 2016 [25]. They proposed various ways to categorize existing methods, but across all categories, one of their key criticisms is fragmentation: there is no comprehensive method covering all phases of risk-driven security decision making. Especially, the first phase, with the purpose of providing an in-depth understanding of a “system, its components and the interdependencies between them, and external factors affecting it” [25], is being neglected. In addition, existing methods provide almost no guidance to connect the different security decision-making phases: if a system is described for security, the results are not directly used for the threat and risk analysis phase, and/or the requirements definition phase.

Another finding of Cherdantseva et al.’s review is the lack of tool support for risk assessment methods, which “could expedite the progress of the domain remarkably” [25]. This is confirmed by Lemaire et al. [26] who reviewed security analysis tools for CPSs in 2017 and concluded that for a comprehensive security analysis, none of the reviewed tools (CSET, ADVISE, CyberSAGE, CySeMoL, and FAST-CPS) are sufficient. In addition, it becomes clear from the analysis that none of the tools support the user all the way through the actual risk-based security decision making. CSET provides checklists and thus supports compliance-based security decision making, and all of the other tools focus on identifying vulnerabilities based on system information the user must provide and sometimes external databases.

Fluchs and Rudolph’s [27,28] 2019 review of risk analysis and systems engineering methods for CPSs confirmed Cherdantseva et al.’s findings. Concerning the lack of a comprehensive method covering all decision-making phases, they identified the need for a comprehensive information model as a basis for such a comprehensive method. Concerning the lack of coverage for the “system understanding” phase, they specifically point out that interconnections between components and humans are mostly not represented in existing system models, and propose the use of combinations of data flow diagrams and use cases known from software engineering as improved system models.

**Goal-driven security decision making** treats security just as functional aspects, where design goals are defined, often taking different stakeholders’ perspectives, and then refined into requirements. It has been pursued to integrate security into software requirements engineering [29,30,31,32,33] and model-based systems engineering [34,35], but not for CPSs. This is probably due to the fact that goal-based engineering is popular in software engineering (so it seemed natural to extend the same principles to software security engineering), but largely unknown in CPS or ICS engineering.

However, one key characteristic of goal-based or agent-based engineering is that perspectives and intentions from different human stakeholders are considered. There has been a lot of research in using concepts to model human intentions (e.g., UML use case diagrams [49] or the Tropos/i* modeling frameworks [36,37]) for security decision making, and the use-case-based approach has also been applied to CPSs, ICSs, and the Internet of Things (IoT) [27,38,39,40].

While goal-driven approaches yield a more complete understanding of the system and its security problems, the inclusion of additional data points such as human stakeholders and their intentions inevitably results in an increased amount of security-relevant information that the security decision maker must process.

**Library-supported security decision making** is an entirely different branch. It can be applied whenever a similar decision has been made before and has been documented in a format that allows re-use. This has the advantage that the necessary security know-how is shifted from those making the security decision to those creating the decision library, and of course re-using existing solutions saves time.

Security patterns are a popular concept for library-supported security decision making. These are generic, re-usable security solutions in a standardized format, often including guidance when to choose which pattern (“pattern system”). Compared to checklists approaches, patterns or related concepts such as solution frames provide more decision-making guidance because they include problem descriptions and indications for choosing the right (generic) solution to a (generic) problem [9,41,42,43]. A specific case of security patterns are the protection profiles used in the Common Criteria standards [44].

Like goal-driven security decision making, this approach has been widely researched for software, but not for CPS. What has been researched in the context of CPS, though, is creating security ontologies. Security ontologies collect knowledge for security-related concepts such as assets, attack scenarios, vulnerabilities, security requirements, and security measures. Some ontologies also include relations between, for example, a risk scenario and recommended security measures, which can be regarded as a way of library-supported security decision making. CPS-related examples can be found in [45,46,47]. In addition, frameworks such as MITRE ATT&CK for ICS [48], including both popular attack techniques and countermeasures, can be regarded as ontologies that can be used for library-supported security decision making.

Library-supported security decision making has a similar weakness as compliance-driven or all checklist-based decision-making methods: The resulting decisions can only be as good as the libraries. As a result, similarly to security requirements checklists, the libraries supporting these methods are ever-growing, increasing the amount of information a security decision maker must digest. Therefore, library-supported decision-making approaches are mostly not feasible without tool support.

There is also a branch of research that addresses the **formal verification and validation of security decisions** once they have been made. These methods require a formal model of the system behavior in terms of continuous and discrete system variables. This formal model can then be simulated, implemented, and evaluated to analyze if the model implementation meets the requirements defined earlier [50,51].

These formal methods are not covered in the state of the art because they do not address the research questions posed in the introduction. The scope of this work is to provide CPS engineers with more guidance to identify security decisions, make them autonomously, and document their rationales; the quality of the decisions and the question of whether the implementation meets the defined requirements are not in scope.

In addition, the necessity to work with formal models is considered too much of an entry barrier for CPS engineers (and even more so for ICS engineers) who have little time for security engineering during design and are usually not familiar with theoretical software engineering methods [8,52].

### 2.3. Summary: Problems of Existing Methods

Summarizing the above sections, Table 2 presents the major problems in state-of-the-art methods for CPS security decision identification and decision making along with their impacts. The column “potential solution” forms a bridge to the concept proposed in this work (Section 3), outlining how it differs from the state of the art.

## 3. Security-by-Design Decisions Method: Workflow for Making Traceable Security Decisions

This chapter outlines a workflow for CPS security decision identification and making. The workflow addresses all problems identified earlier. The innovative components that facilitate the workflow are:

- **Function-based security parameter libraries** for decision identification;
- **Function-based security diagrams**, and in combination with an underlying
- **Data model and tool**, these diagrams guide decision making and enable traceable security decisions to be made.

Both libraries and diagrams are function-based. We use “function” as a combination of the function concept as defined in systems engineering [53,54] and the UML use case concept [49]: While a use case can be understood as the intention that a (human) user has when interacting with a system, a function involves anything that the system needs in order to implement a use case, but may also include functionality not directed toward any visible use case. This difference matters when modeling either concept: For modeling a use case, the system is regarded as a black box. For modeling functions, the system’s internal functionality is important.

The advantages of this hybrid function concept as a basis for a security-by-design method are: (1) it is a good way to bundle security-relevant information: a system’s internal functionality, but also a system’s users and intended use; (2) it is suitable for modeling a system early in the design phase without anticipating implementation details. For details on the function concept, refer to [10,27,40]. Function examples are provided throughout the remainder of this paper.

### 3.1. Workflow Overview

**Figure 1:**
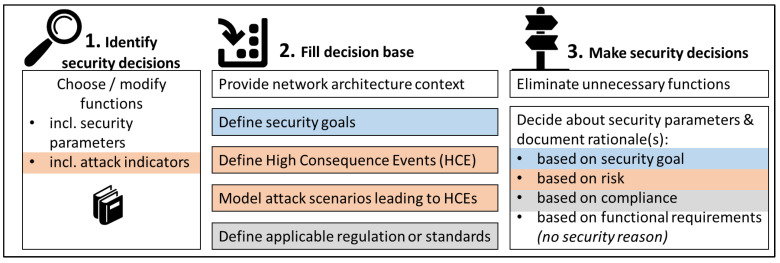
Security-by-design decisions method: Workflow for making traceable security decisions supported by function-based security libraries and function-based security diagrams, a security data model, and a software tool. The workflow accommodates different security decision-making paths: risk-driven (orange), goal-driven (blue), and compliance-driven (grey).

The security-by-design decisions workflow is summarized in Figure 1. There are three general workflow steps (1. identify security decisions, 2. fill decision base, 3. make security decisions), but within these steps, the concept provides a decision-making framework rather than a strict workflow: In steps 2 and 3, all activities are optional. If they are needed depends on the decision-making path that is chosen for a given project or a given security decision.

The workflow accommodates all four security decision-making paths introduced in Section 2 and [10], but none are mandatory. Identification of security decisions is library-supported (but it does work without libraries if desired). Security decisions can be made following a goal-driven (marked blue in Figure 1), risk-driven (orange), and/or compliance-driven path (grey), depending on the resources provided in the decision base. Regardless of the chosen decision-making path, the rationale for each decision is documented, enabling the traceability of decisions. This is enabled through diagram and tool support for the workflow.

It is important to note that the aim of the method is to provide decision makers with all information to make their security decisions in a systematic, informed way, and make sure decisions are traceable (i.e., decision rationales are documented).

The method does *not* recommend how security decisions be made, and neither does it require following any specific decision-making path. Decisions can be made following multiple paths at the same time. Often, the result is simply multiple rationales for a single decision (e.g., disabling remote support is needed for compliance reasons and because the risk is deemed too high), but of course, two decision-making paths may also result in conflicts of interest. In these cases, it is upon the decision maker to make (and again: document!) the decision.

This is especially true if a security decision is made based on functional requirements, which is mostly the case for decisions that are not ideal or even detrimental from a security perspective. However, it must be acknowledged that security is not an end in itself and often requires a compromise between price, usability, feasibility, and security. Nevertheless, there is a value in being aware of and documenting that a non-ideal security decision has been made.

In the following, the different paths through the workflow are described in more detail.

**Step 1: Identify security decisions**

This first workflow step is the same for all paths. From a library, security decision makers choose all functions that apply to the system under consideration. Functions may also be modified if needed.

*Example: From the library category “engineering”, the functions “update of PLC logic”, “test and debugging of PLC logic“, and “sensor calibration” are selected.*

There is a set of diagrams for each function (see Section 3.3 for details), and each function contains one or more security parameters, each of which marks a security decision. Thus, choosing applicable library functions is equivalent to identifying security decisions. A security parameter has the form of a title and several possible values the parameter can assume: *title: {value 1, value 2, value 3, …}.* The function and security parameter libraries are described in detail in Section 3.2.

*Example: For the function “update of PLC logic”, security parameters are “code block protection: {enabled, disabled}”, “change of operating modes: {key switch, password}, “updating logic during operations: {enabled, disabled}, or “integrity protection of PLC logic: {none, checksum, hash}”.*

**Step 2: Fill decision base**

Now, a security decision base is created and filled with everything that may help make the security decisions that were identified before. All items are optional, and their existence relies on the decision-making paths that an organization chooses to follow:

- **Goal-driven decision making** (marked blue in Figure 1): For goal-driven decisions, the security decision base is filled with security goals for specific functions or function components or the overall systems. Security goals are often composed of (combinations of) confidentiality, integrity, and availability of certain functions, components, or pieces of information, but they can also include reliability or compliance aspects [10]. Goals can also be hierarchically organized into sub-goals to allow the break-down of high-level goals into more specific ones.
  *Example: For a chemical production plant, a security goal could be “stable pressure in reactor” with a sub-goal “integrity of output value for pump P”.*
- **Risk-driven decision making** (marked red in Figure 1): For risk-driven decision making, it can make sense to define High-Consequence Events (HCEs)—events that would make for a really bad day at the organization and that must be prevented [10,55]. In addition, all kinds of attack scenarios potentially leading to HCEs are a meaningful addition to the security decision base because they provide more detailed indicators on how to prevent HCEs. Attack scenarios can be function-specific, detailing how a specific function may be exploited to cause a specific HCE.
  Security parameters contain some built-in guidance for constructing attack scenarios: parameter values that may be used in an attack are marked as attack indicators in the library. See Section 3.2 for details.
  Attack diagrams provide guidance by overlaying all relevant information for modeling attack scenarios onto function diagrams. See Section 3.3 for details.
  *Example: For the same chemical production plant, a HCE could be “reactor explodes”. For the function “update of PLC logic”, an imaginable attack scenario would be sending a spearphishing attachment to a PLC engineer to gain access to her programming device, exploit a vulnerability allowing user execution on that device, and ultimately modify the PLC’s logic to cause overpressure in the reactor.*
- **Compliance-driven decision making** (marked grey in Figure 1): For compliance-driven decision making, the decision base simply needs to be filled with the regulations, laws, or standards affecting security that should be complied with. These can be international, national, or even internal company regulations.
  *Example: For the chemical plant, it is determined that the company-internal policy “automation security guideline ABC” needs to be complied with. It has eleven paragraphs that may each be referenced separately.*

**Step 3: Make security decisions**

Finally, the decisions identified in step 1 are made. More specifically, making a security decision means deciding to set a security parameter to one of its possible values. Alternatively, the decision to eliminate a system function—along with all the entities it is composed of and all security parameters—is a valid security decision.

All supporting facts collected in the decision base in step 2 can now be built upon to make an informed security decision and to document a rationale for each decision. Because the security decision base may have grown considerably large at this point, security diagrams play a major role for decision making because they efficiently present the information needed to make the security decisions (see Section 3.2 for details). In addition, the decision maker is guided through the decisions function by function, which reduces complexity for each decision. Again, all decision-making paths are supported:

- **Goal-driven decision making** (marked blue in Figure 1): For goal-driven decision making, the security parameter contributes to fulfilling a security goal defined before. This security goal forms the rationale for the security decision.
  *Example: for the security parameter selection “integrity protection of PLC logic: hash”, a goal-driven rationale could be “stable pressure in reactor R”, or more specifically “integrity of output values for pump P”.*
- **Risk-driven decision making** (marked red in Figure 1): For risk-driven decision making, the security parameter helps mitigate a risk, or to be more precise, it helps to reduce the impact or likelihood of a high-consequence event (HCE) occurring; thus, the addressed HCE serves as a rationale for the risk-driven security decision. If attack scenarios that include one of the security parameter’s attack indicators have been defined, these may be referenced in the rationale as well.
  *Example: for the same security parameter selection “integrity protection of PLC logic: hash”, a risk-driven rationale could be that the parameter value “integrity protection of PLC logic: none” is used in a potential attack scenario leading to the HCE “reactor explodes”.*
- **Compliance-driven decision making** (marked grey in Figure 1): For compliance-driven decisions, the security parameter helps comply with a specific regulation or standard. As a rationale, it is sufficient to reference the relevant regulation or standard.
  *Example: for the security parameter selection “CVE-2023-001: patched”, a compliance-driven rationale could be “national critical infrastructure protection directive, §1” (assuming that, in the example country, patching certain vulnerabilities is mandated in critical infrastructure regulation).*

Thus, each decision (i.e., each selected security parameter value) has at least one rationale—a goal for a goal-driven decision, an HCE and maybe an attack scenario for a risk-driven decision, and a regulation or standards for a compliance-driven decision. For some decisions, several rationales may be combined, so a parameter value may have been selected to both mitigate a risk and to comply with regulation.

Lastly, there is a fourth type of security decision rationale:

- **Functional-requirement-driven decision making:** Sometimes, a security decision is made for no security reason, but because the need to fulfill a functional requirement has taken the security parameter out of the solution space available to security decision makers.
  Although it might sound counter-intuitive to call a decision a “security decision” even though it has been made for no security reason at all, it should be remembered that it is a security decision because it influences the system’s security posture, not because it was made for security reasons. Maybe even more than for security decisions actually made for security reasons, it matters to be aware of these decisions, because it means the overall security posture is (often negatively) impacted by decisions made outside the security domain.
  *Example: for the security parameter selection “updating logic during operations: enabled”, a functional-requirement-based rationale could be “enable on-line program changes to accommodate for fast adaptation of modular plants”.*

### 3.2. Function-Based Security Parameter Libraries

The security parameter library has the main purpose of making decision identification fast and easy for decision makers who neither have the time nor security expertise. Therefore, each security parameter is tied to one or more functions, or to be more specific, to one or more function entities. Therefore, the security parameter library really is composed of four related libraries: security parameters, functions, entities (including humans), and protocols.

For security decision identification, the decision maker does not select security parameters, but functions that the system under consideration is intended to fulfill (see an excerpt of library function categories and functions in each category in Table 3). The security parameters are tied to the function entities, so they are gathered automatically once all relevant functions are selected.

*Example: The security parameter “logic update during operations” is tied to the entities “PLC” and “Safety PLC”. Thus, it is identified as a security decision whenever a function containing a PLC or safety PLC is selected in workflow step 1.*

Security parameters in the library have fixed attributes. Security parameter attributes are displayed in Table 4, using the parameter “logic update during operations” as an example.

The function-based security parameter library concept addresses the following of the previously identified problems:

**Problem 1: “Undercover” security decisions:** The security parameter library serves the purpose of facilitating a more complete security decision identification than usual security requirements checklists. Security parameters make security decisions visible, including configuration details that may not seem security-relevant at first sight or “insecure by design” features.

In addition, security parameters are unlike security requirements because they do not have a required value. They have two or more possible values and neutrally inform the decision maker about the security implications of each of these values.

**Problem 2: Lack of comprehensive guidance for security decision making:** As described above, the security parameter library is function-based, so the decision maker does not have to select from a list of security parameters, but simply which functions the system under consideration is intended to fulfill. That way, the decision maker is guided through the decision identification process by answering one simple, not-at-all security-related question: What is my system intended to do?

Beyond providing guidance for decision identification, the libraries contribute to guiding the decision maker through decision making because security parameters can be decided upon function by function and because the security parameters contain built-in guidance: explanations why they are security-relevant and which possible parameter values could be used in a security attack (attack indicators).

**Problem 3: Shortcomings in system understanding:** Because selecting security parameters takes place through selecting functions, a basis for system understanding is created on the fly. While a network map may also be created, functions contain more information than only a network map: interactions/data flows between components, human “components”, and the intention of the system components and interactions.

**General problem 1: CPS engineers do not have security expertise:** Security parameter libraries encapsulate the security knowledge that decision makers are lacking. They contain the information regarding which of the many system aspects have an impact on security, why that is, and which possible parameter values have which security implication.

**General problem 2: CPS engineers do not have time for security:** Libraries in general are big timesavers because instead of creating information, decision makers simply have to choose from existing information, which can be carried out much faster. Because the security parameter libraries are function-based, time is saved not only for security decision identification, but also for system modeling and understanding, which is a precondition to informed security decision making.

### 3.3. Function-Based Security Diagrams

In Ref. [56], the authors have analyzed in detail why and how visual representations are beneficial for security decision making. These are the two main reasons:

First, the security information base can grow to a considerable size, and security decision makers cannot be experts for all the information they have to digest because it stems from a broad range of domains (hardware/software engineering, process engineering, network engineering, potentially safety engineering…). Research has proven that visual representations are superior to other representations in scenarios where lots of information need to be communicated to non-experts, because large portions of visualizations are consumed subconsciously and thus highly efficiently [57,58,59,60].

Second, much of the information relevant for security decision making is difficult to convey text-only. Especially for functions and network maps, there are core pieces of information that cannot be adequately presented in texts or tables: location and relations of the entities. However, for the proposed security decision-making workflow, functions (and networks) are of fundamental importance in every workflow step.

**Figure 2:**
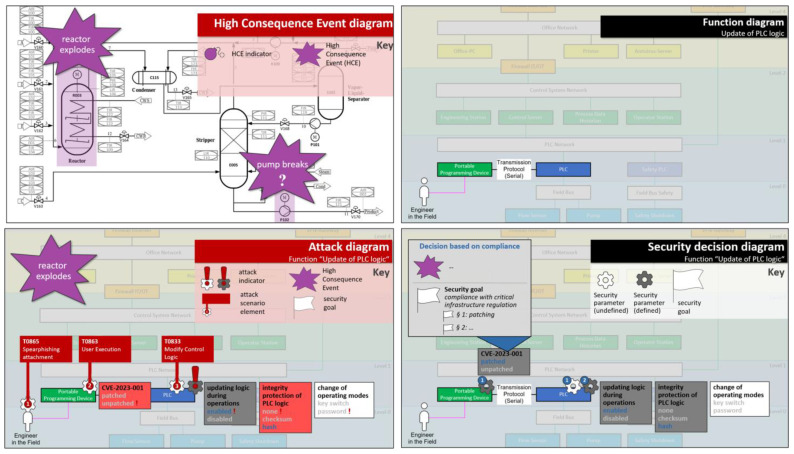
Four proposed diagram types for security decision making [56]. All diagrams except for the High-Consequence Event diagram are function-based: they use a function drawing as their base and layer other relevant security concepts on top. The security decision diagram is designed to facilitate decision making, while the other three diagram types help in filling the decision base (see Table 5 for details). The numbers in blue circles in the security decision diagram indicate the number of security parameters for the respective entity. The numbers in red circles in the security attack diagram indicate the sequence of events in the attack scenario.

Again in [56], the authors have carved out requirements and main pillars for useful visualizations in the context of security decision making. Four diagram types were developed that fulfill these requirements: High-consequence event diagrams, function diagrams, security decision diagrams, and attack diagrams (see Figure 2). The security decision diagram is designed to facilitate decision making, while the other three diagram types help in filling the decision base. Table 5 summarizes how each of the diagram types is used in the security decision-making workflow.

*Example: The function “update of PLC logic” is deemed relevant for the system under consideration. The library function shows two ways of updating PLC logic, one over the network, and one using a serial connection between the programming device and PLC. For the system under consideration, the function drawing is modified to display only the serial option.*

*Next, the security decision base is filled. The high-consequence event (HCE) “reactor explodes” is selected from the high-consequence event diagram because abusing the function “update of PLC logic” could potentially lead to this HCE. An attack diagram is opened to show all potential attack points for this function, and some of them are used to model an attack scenario leading to the HCE. Back in the security decision diagram, a security goal is recorded—critical infrastructure regulations need to be complied with.*

*The security decision diagram shows four security parameters to be decided for this function. To decide, the security decision base is consulted. All relevant information can be layered on top of the security decision diagram: the security goals, the attack scenario, and which attack points have been used in the attack scenario. Based on this information, it is decided that CVE-2023-001 is to be patched (because of the compliance security goal and because the CVE plays a major role in the modeled attack scenario). In addition, the attack scenario suggests that it matters to protect the integrity of PLC logic, so the “hash” option is selected for this parameter. It may also help prevent the attack scenario if the update of logic during operations would be disabled, but this is not an option, because it is a functional requirement—this security decision has already been made, as the security decision diagram shows (also see Figure 5).*

**Figure 3:**
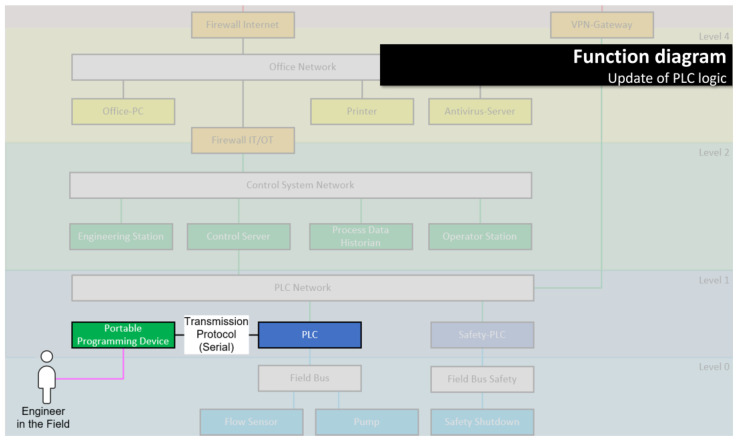
Function diagram mockup for example function “update of PLC logic” in the context of an exemplary network model.

**Figure 4:**
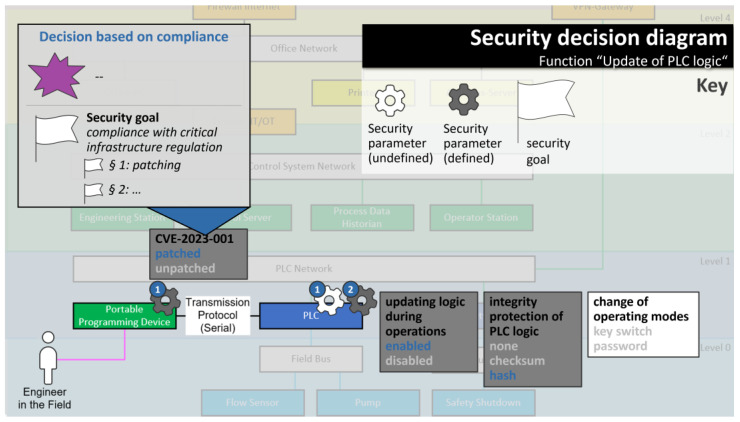
Decision diagram mockup for example function “update of PLC logic” in the context of an exemplary network model. The numbers in blue circles indicate the number of security parameters for the respective entity.

**Figure 5:**
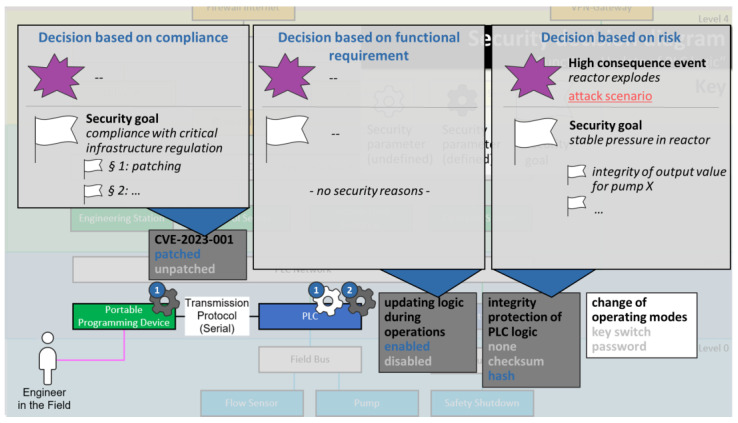
Decision diagram including decision rationales for example function “update of PLC logic”. The numbers in blue circles indicate the number of security parameters for the respective entity.

Examples for the function and security decision diagrams are given in Figure 3, Figure 4 and Figure 5 (the use of the other two diagram types is optional). All examples are mockups that were used for the requirement definition for the software demonstrator (see Section 3.4).

The function-based security diagram concept addresses the following problems identified earlier:

**Regarding problem 2: Lack of comprehensive guidance for security decision making:** For the security decision base, it helps to have all relevant information available, but it can grow quite large and an unstructured pile of information does not exactly qualify as decision-making guidance. The four diagram types help to only present the relevant information needed for each activity in the decision-making workflow.

Additionally, the fact that diagrams are function-based reduces the complexity because decision makers can be walked through all their decisions function by function instead of looking at an overwhelmingly large number of open decisions and relevant information.

**Regarding problem 3: Shortcomings in system understanding:** The most important means for system understanding in the proposed workflow is the function concept, and, as discussed earlier, functions cannot be completely represented without diagrams.

**Regarding problem 5: Increasing amount of information to be digested**: Diagrams, as mentioned earlier, are superior to other representations in scenarios where lots of information need to be communicated [56,57,58,59,60].

**Regarding general problem 1: CPS engineers do not have security expertise:** Diagrams, as mentioned earlier, are superior to other representations in scenarios where information needs to be communicated to non-experts [56,57,58,59,60].

**Regarding general problem 2: CPS engineers do not have time for security:** As mentioned earlier, communicating information in diagrams saves times because large portions of visualizations are consumed subconsciously and thus highly efficiently [56,57,58,59,60].

### 3.4. Workflow Support by Security Data Model and Tool

The proposed decision-making method is implemented in a software demonstrator. The demonstrator is built as a web application with a React frontend based on Javascript and a Flask backend based on Python. For data storage, a Neo4j graph database is used. Therefore, the data model is graph-based as well. It contains all concepts previously introduced in this chapter: functions, entities, protocols, high-consequence events, security parameters, attack indicators, attack scenarios, security goals, and standards/regulations. Most importantly, the software demonstrator contains the function and security parameter libraries and can display interactive versions of all security diagrams introduced in Section 3.3. “Interactive” means that all parts of the diagrams can be edited, and additional decision base information (security parameters, attack points, security goals, high-consequence events, standards, etc.) can be dynamically shown, hidden, and filtered based on keywords. In addition, it guides the user through the decision-making workflow displayed in Figure 1.

To enable the technology-independent exchange of engineering data, the demonstrator has an import/export interface to an AutomationML information model representing all of the above concepts. For details on the AutomationML model, see [61]. In addition, a corresponding UML information model is currently being created at NAMUR WG 1.3 (Information models).

The security data model and tool address the following problems identified earlier:

**Regarding problem 2: Lack of comprehensive guidance for security decision making:** A tool does not only make a workflow more efficient as it drastically reduces documentation efforts; it can also serve as guidance.

**Regarding problem 4: Lack of security decision traceability:** The true reason why the proposed workflow enables decision traceability is the carefully selected concepts (functions, security parameters, HCEs, security goals, attack indicators, attack scenarios, regulations), which cover all possible security decision-making paths [10]. However, traceability does not come from the isolated use of any of these concepts. To the contrary, each decision must be tied to a number of concepts to provide a precise rationale to why it was made.

The rationale would be complicated and tiresome to document (and a nightmare to maintain!) without a data model and tool capturing all additions to the decision base and all decisions made including their relation to decision base elements while the decision maker is working.

**Regarding problem 6: Lack of tool support.**

**Regarding general problem 1: CPS engineers do not have security expertise:** As an addition to Problem 2 (lack of guidance): especially if a workflow is not exactly daily business for the users, a tool can help remember the workflow steps.

**Regarding general problem 2: CPS engineers do not have time for security:** As explained in Chapter 3.3, diagrams are an indispensable prerequisite for enabling the security decision-making workflow. A big benefit of the proposed diagram concept is the selection of only a fraction of the entire security decision-base to be displayed at once to enable focused decision making. However, this flexibility increases the number of different diagrams that are needed.

Without a data model and tool that saves all decisions and the entire decision base as a “single source of truth” from which the diagrams are generated dynamically as needed, the workflow would not be realistic in any context, and even less so in a context where time is very limited to begin with.

### 3.5. Limitations and Open Issues

#### 3.5.1. Library Maintenance

As for all libraries, maintaining security parameter libraries is a challenge. There are two advantages that make maintenance easier:

First, functions change little over time—it will always be needed to update PLC logic in some way or the other. Thus, for the function part of the library, little maintenance is expected.

Second, security parameters are mostly independent of the dynamic cybersecurity threat landscape because they affect design decisions that are made anyway. What changes with the threat landscape is the decision base for the risk-driven decision path: New threat scenarios may arise or their likelihood may increase, and additional parameter values may turn into attack points. This potentially changes how the security decision is made, but it mostly does not add new decisions, because the design options remain the same. The only exception is the security parameters that explicitly contain vulnerabilities, but these can relatively easily be kept up-to-date by querying CVE databases (if this is not carried out as part of vulnerability management anyway).

The library maintenance concept includes not having one centralistic library but having organizations build their own libraries. The authors intend to collaborate with CPS manufacturers in the future, because they have the knowledge required to build the security libraries for their components. With upcoming regulations such as the EU Cyber Resilience Act [2], manufacturers will be incentivized to transparently document their components’ security capabilities and recommended security configurations anyway, and maintaining a security-parameter library along with recommended parameter values can be an efficient way to do so.

#### 3.5.2. Additional Security Decisions (Lifecycle Decisions)

As described in Section 3.2, decision identification in the presented work is completely based on the function-based security parameter library. This implies that each decision must be expressed as a security parameter and tied to one of the entities that the functions are composed of; otherwise, it would not be identified in workflow step 1.

The authors are aware that this limits the decisions that can be identified. Not all security decisions can be tied to a function or a specific technical or human entity. In fact, next to these **function-based security decisions**, a second group of security decisions called **lifecycle security decisions** is included in the security parameter library as additional security parameter attributes.

Lifecycle security decisions are not included in this work, because they have not been included in validation yet. They are called lifecycle security decisions because they can be tied to specific points in the systems’ lifecycle beyond design (including the operations phase) for the system under consideration:

- **Procurement:** Decision of which security parameters should be included in procurement as SHALL or SHOULD criteria;
- **Audits:** Decision of which security parameters shall be audited (and how) in factory acceptance tests (FATs), site acceptance tests (SATs), or other tests/audits;
- **Operations (monitoring):** Decision of which security parameters should be visualized on an HMI or alarmed;
- **Operations (procedures):** Decision of which security parameters need organizational procedures to be adhered to or are inputs to specific procedures.

## 4. Validation

### 4.1. Validation Setup

The validation of the security-by-design decisions concept introduced in this paper was carried out in a case study/technical action research: An already completed engineering project at HIMA, a specialist for safety-related automation solutions, was conducted again (“validation project”), but this time using the proposed security-by-design decision making concept implemented as a software demonstrator, as described in Section 3.4.

The test persons, i.e., the security decision makers, were HIMA engineers familiar with the original project. They were not familiar with the proposed security decision-making method before the validation. The HIMA engineers were responsible for integrating HIMA products into the plant owner’s plants; they were not the product designers.

The researchers that developed the methodology were actively moderating, but only to make sure the methodology was being adhered to. They did not comment on or suggest decisions in any of the three workflow steps outlined in Section 3.1. The validation was conducted in three workshops over the course of one week.

The subject of the validation project was the review of a legacy automation system for a plant that produces chloroformates. In the course of digitizing the automation system, availability and security requirements needed to be reviewed. The automation system scope extended over various HIMA functional safety controllers, OPC servers, and workstations as well as network infrastructure and signal interfaces to third-party control systems.

### 4.2. Validation Method, Questions, and Metrics

The validation was planned following the Design Science Methodology by Wieringa [62]. The validation goal was to answer the three research questions (summarized as decision identification, decision making, decision tracing) from Section 1. For being able to measure the validation’s success, the research questions were concretized into the measurable validation questions along with a mix of quantitative and qualitative metrics for answering them (Table 6). A question for decision re-use was added to explicitly measure the validity of the function-based security parameter libraries. For the quantitative metrics, the researchers took counts during the validation projects. Comparisons to the original project were made either based on the past project’s documentation or by asking the test persons about differences. For the qualitative metrics, feedback was collected from the test persons during an interview directly after the validation project.

### 4.3. Validation Setup Limitations

The strength of the validation setup is that a real project from a real company could be used as a case study. The major limitation is that the sample size was limited—it was only one project at one organization (HIMA) in one country (Germany).

However, the project was not accompanied with the new methodology in real time but revisited in a “lab environment”. This has both advantages and disadvantages: There was less time pressure, so the test persons could take the time to really understand the methodology to be validated. In addition, software and usability issues caused by the software demonstrator being in an early stage of development did not distort the results. However, in reality, some time pressure will always be present and one of the underlying assumptions in the research questions was that engineers had limited time. To address this issue, limited time was allocated for the validation (and time limitations were communicated up-front). Additionally, the validation period was set to only one week.

Another limitation is that the organization who conducted the validation project, HIMA, is a specialist for safety-related automation solutions with a very limited product range (safety controllers). As pointed out in Section 1, the proposed methodology addresses engineers at asset owners, integrators, and component manufacturers, so at least the asset owner view is missing in the validation setup.

The validation project was an authentic engineering project with a broad range of used HIMA products, which was good. However, it had a specific security focus: the security of already existing systems and their network architecture were to be improved. This made it more difficult to “re-evaluate” the project with the security-by-design decisions method, because the project itself was already a security “re-evaluation”. The advantage of this setup is that the bar for identifying and making additional security decisions was quite high, so where additional security decisions were identified, it could be regarded a real success for the new method.

The test persons who conducted the validation project using the new method were also part of the team who conducted the original project (without the new methodology). While this has the advantage of them knowing the project well and being able to compare the results with and without the new methodology, it also bears the risk of them being biased toward the decisions they made in the original projects or being reluctant to admit that they may have overlooked something or made a security decision that was not ideal. In addition, the test persons did not completely fit the target audience for the security-by-design decisions methodology (engineers with little time for security and little security expertise), because they were skilled in security.

For decision identification, it would have been interesting to observe if test persons arrive at the same results independently. However, only one group of test persons was available.

### 4.4. Validation Results

The results for all quantifiable validation metrics are summarized in Table 5 and, alongside the qualitative results, explained below.

**Figure 6:**
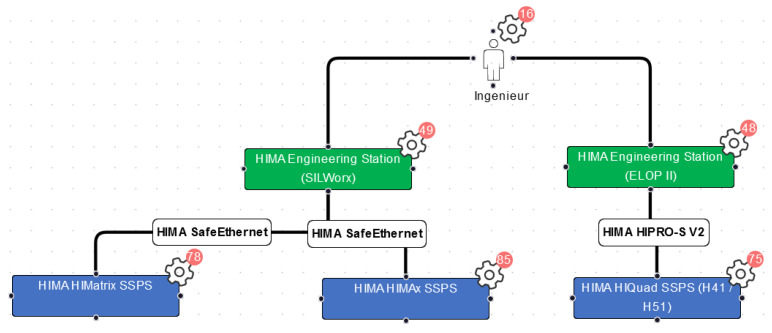
Modified library function “Engineering of safety systems”. The cogwheels indicate identified security decisions (security parameters). The red circles indicate the number of security parameters for each entity.

**Figure 7:**
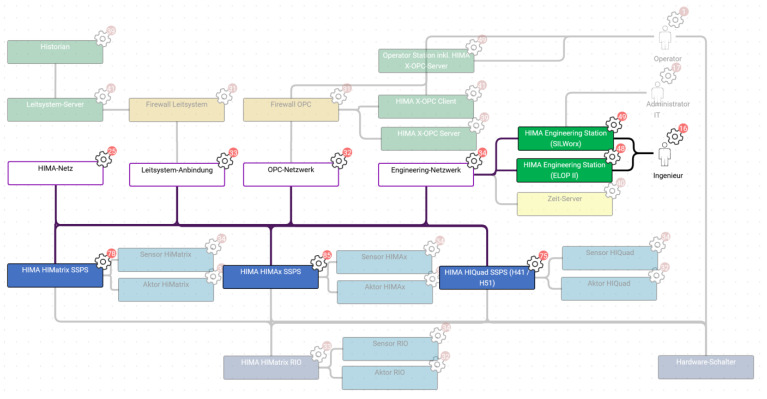
Function “Engineering of safety systems” displayed in network architecture context. Cogwheels indicate identified security decisions (security parameters). The red circles indicate the number of security parameters for each entity.

**Decision identification** was performed by selecting functions from the function library and modifying them to fit the validation project (see Figure 6 for an example). Afterward, the network architecture (Figure 7) was modeled by combining all entities from the functions. The selection and modification and architecture modeling took about 3.5 h. As a result, 148 security parameters associated with the modeled functions were identified. Among those were 27 that contained decisions that were not identified in the original project. The test persons stated that the resulting system model reflected their system well.

Switching perspectives between function view (Figure 6) and network architecture view (Figure 7) yielded interesting results. From the function drawings, it becomes apparent which entities need to communicate (and how) to fulfill a certain purpose. In the network architecture drawing, the information from the function is set into context—with other, uninvolved entities, network segments, maybe security components such as firewalls—and it becomes obvious if one of the interactions needed for the functions becomes problematic within the network architecture—for example, because network segments need to be connected that were supposed to be isolated.

This occurred for at least one function (“archiving of notifications”) in the validation project: the communication path in the function seemed clear, but in the proposed network architecture, it became clear that there was no connection intended between two of the involved entities.

After decision identification, the **decision base** was filled in one hour. Three high-consequence events (HCEs), ten attack scenarios, eleven security goals, and six standards or regulations were created to guide decision making.

**Decision making** was limited by the available workshop time. Four hours were enough time to go through 74 decisions. A total of 13 of those did not apply to the validation project, but the remaining 61 decisions were made, resulting in about 15 decisions made per hour.

Of these 61 decisions, 27 (44%) were additional decisions. There were 4 completely new decisions that had not been considered in the original project at all, addressing where and how passwords are saved, how the legitimacy of software is checked before installation, and how tampering with the IP to ARP assignment is prevented. The remaining 23 additional decisions were made in the original project, but without considering their security impact at all. These decisions often affected topics that are not typically associated with security: sensor redundancies, alarm times, alarm priorities, and alarm texts for potentially security-relevant alarms, parallel sessions, bridging and forcing of signals, OSI layer 1–3 signal processing choices, how new software is deployed, use of shared hosts, etc.

A quantity of 4 out of the 61 decisions were changed compared to the original project. However, the significance of this number is limited because the test persons were the same engineers who also carried out the original project, so they (understandably) rather explained and justified their decisions made in the original project than change them.

There were no decisions that could not be made at all, and in the follow-up interview, the test persons said they were mostly the right ones to make the decisions. However, for seven decisions, they stated that either the asset owner or product designers were the ones that ultimately had to decide.

The test persons stated that as additional information for decision making, they would have liked to include more details in the architecture drawing, e.g., zones and conduits according to IEC 62443-3-2 [63]. In addition, they said an overview of the resulting compliance with selected standards for their decision making would have been helpful. They also suggested a feature to postpone a decision to decide later, and they said they would have wished for more guidance regarding the sequence in which they made the decisions.

**Decision rationales** were given for all decisions—there were no decisions for which no rationale could be selected. Most decisions (57%) were made based on functional requirements (or functional restrictions, when some options were not technically feasible). In other words, most security decisions were not made for security reasons. The number of risk-based and goal-based decisions was roughly the same (36%/30%). These two rationales were often given at the same time because risks and goals are often closely coupled. For example, if a decision is made to meet the goal “Access to components only for authorized persons”, it likely also mitigates the attack scenario “forcing of a sensor value”. Likewise, a decision that contributes to the goal “engineering station cannot be abused maliciously”, likely also prevents the attack scenario “manipulation of safety PLC logic”.

Only for 15% of decisions, test persons selected a compliance-based rationale. This was an especially interesting result because they beforehand expressed the feeling that the majority of their security decision making was for compliance reasons.

In the interview, test persons estimated that they could use the decision rationales for communication toward clients or management if there were enough accompanying details—i.e., the exact risk, goal, or standard requirement for risk-driven, goal-driven, or compliance-driven decisions.

Regarding **decision re-use**, the re-use of functions and security parameters, re-use of decision base elements (HCEs, security goals, attack scenarios, and standards), and re-use of made decisions and their rationales must be differentiated between.

For functions, a relatively small portion of library functions was used (13%), but this was no surprise, since HIMA is also a manufacturer for safety controllers, and therefore all parts of the library containing ordinary PLCs, control systems, or office-related activities such as printing did not apply at all. Of the selected library functions, a relatively large percentage (64%) was modified to fit HIMA’s specific architecture and protocols, but no function had to be newly created, indicating a good completeness of the function library.

However, although only 13% of functions were selected from the library, 86% of the library’s security parameters were deemed applicable. This is due to the fact that many security parameters from the library are assigned to multiple functions and entities.

Test persons estimated that the resulting function and architecture models would be re-usable for future projects, because HIMA as a component manufacturer obviously tends to use the same components and protocols for the same reasons across many projects. The same was true for the decision base. The HCEs, attack scenarios, and security goals were deemed so universal that they could probably be completely copied for the next project. Regarding the security decisions and their rationales, test persons said the potential for re-usability would be highly dependent on the client, the client requirements, the automated process (i.e., the client industry), and the applying standards.

As **overall feedback**, the test persons stated that the biggest advantages they saw were hints to security-relevant aspects they had not considered before.

They were both experienced engineers who knew their systems and their security features well and said that while, for them personally, the method did not make as much of a difference, they imagined it valuable to systematically document know-how, allowing less experienced colleagues to make security decisions as well without needing to consult the rare specialists. In addition, they stressed that the export of security decision data to pdf and ideally also machine-readable formats was important, and they wished that there was an “update service” and/or a trigger for revising security decisions.

## 5. Discussion

In the following, the key findings from the validation are discussed.

The first observation is that visualizations have generally been perceived as one of the method’s strengths. The function library seemed to have a good quality, because no additional functions needed to be created to fully model the scope of the validation project. Although 64% of the functions had to be adjusted, the library-based modeling was efficient because adjusting an existing model is faster than creating a new one.

In addition, the comparison of interplay of function and architecture drawings even yielded completely new insights because functionally needed interactions could be compared with those architecturally intended.

When asked which additional feature they would prefer, the test persons chose the additional modeling of attack scenarios, stating that they saw a **high value in visualization**.

The strength of the strongly visualized parts of the methods correlates with some weaknesses where long lists of information with little visualization needed to be consumed: the security parameters. The overwhelming number of 148 security decisions in the form of 148 security parameters was difficult to digest in the list-based form that it currently has. Although the authors tried to bring structure to the list by assigning parameters to modeled entities, this did not add much structure, because most parameters applied to a lot of different entities: although only 13% of library functions were deemed applicable, 86% of library parameters were attached to these 13% of library functions. Consequently, the test persons rightfully complained that there was too little guidance regarding the sequence of security decision making, and the visualizations that helped identify decisions were put to too little use for decision making. The necessary **improvements of the structure of and navigation through security parameters** are among the biggest learnings from the validation.

The rationales for decisions made held a surprise: By far, **the highest number of security decisions was not made for security reasons**, but because of functional requirements and/or restrictions. This correlates with the relatively high percentage (44%) of decisions that were newly identified (as security decisions) using the methodology.

In combination with the test persons’ statement that one of the most important advantages of the methodology was helping them to think about aspects they may not have covered so far, this is a strong encouragement to lay an emphasis on adding security parameters from engineering domains to the library that may not look security-relevant at first sight. These are exactly the “undercover” security decisions introduced in Section 2.

Lifting the cover from undercover security decisions is obviously important if they are otherwise made in a way that is detrimental to security, but it also matters if they were otherwise unconsciously made in a way that is beneficial for security, even if the decision is good: if it is not explicitly a security decision, it cannot be communicated as such to clients or fellow engineers, and if it is ever changed, the security impact will be overlooked.

Additionally, the validation results call for a **more diverse understanding of security decisions**. While, so far, the method equates a security decision with setting a security parameter, the validation made clear that architectural decisions such as adding network segments or security components such as firewalls, data diodes, or other security appliances such as intrusion detection systems should explicitly be treated as security decisions, including the traceability of their rationales.

The test persons also indicated an interest in the lifecycle security decisions introduced in Section 3.5.2, especially those covering security alarms and security acceptance tests.

Lastly, it became clear from the validation that the method so far covers security-by-design aspects for integrators and asset owners, but it requires a whole different level of modeling and security parameters to also cover **security by design for product developers**. To cover security for products, function and security parameter libraries for intra-component entities such as CPUs, RAM, and clocks are needed. These product design libraries should ideally be tested in another round of validation specifically targeting product developers.

## 6. Conclusions and Outlook

The security-by-design decisions method presented in this paper has proven to help CPS engineers autonomously identify and make security decisions that they may not have made (consciously) without it—fast and with little security expertise. This means that more of the CPS experts with little time, but also more people with a lower level of CPS and/or security expertise, can contribute to addressing security issues in products, automated plants, and critical infrastructures before users put a hand on the systems (“security by design”).

The problem analysis in this paper has shown that the major problems with security decision making for CPSs are that non-obvious security decisions are overlooked, that there is no comprehensive guidance for making security decisions during system design, that existing methods do not reflect the multitude of reasons why security decisions are made in practice, that the system understanding phase and the traceability of security decisions are especially underrepresented in state-of-the art methods, and that many improvements of CPS security by design correlate with an increasing amount of security-relevant information to be digested, which can only be performed efficiently if there is tool support.

The presented concept addresses these problems by providing a software demonstrator that guides security decision makers through the decision-making process and supports them with interactive diagrams, pre-filled security libraries, and an automatically populated data model to store all decisions and their rationales.

One of the biggest proven strengths of the method is to draw attention to security decisions that may otherwise not have been identified as such. Each consciously made security decision that may otherwise have been overlooked means that there is one fewer potential vulnerability; one fewer potential vulnerability that end users need to be notified of, assess, and fix; one potential vulnerability that—if not fixed—could result in industry incidents harming people or the environment, or causing outages in critical infrastructures such as food, water, or power supply.

One of the biggest challenges of the method is to adjust the level of detail for the security libraries so that they are still maintainable, but specific enough to be useful, and to present the identified security decisions in a way that is intuitive and efficient to navigate for the security decision makers without having to comb through long lists. In addition, library maintenance is a challenge. The authors intend to collaborate with CPS manufacturers in the future, because they have the knowledge required to build the security libraries for their components. With upcoming regulations such as the EU Cyber Resilience Act [2], manufacturers will be incentivized to transparently document their components’ security capabilities and recommended security configurations anyway, and maintaining a security-parameter library along with recommended parameter values can be an efficient way to do so.

The software demonstrator used to validate the presented method was an early version that did not yet cover all planned parts of the method. In future research, it will be completed, adding extra modules to model attack scenarios and help with the timing of security decisions within existing engineering workflows (see [64,65,66] for groundwork).

In addition, the key findings from the validation described in this paper will be addressed: additional decision types will be introduced, and more guidance for navigating through all identified security decisions will be provided.

While the validation was so far carried out with integrators, additional rounds of validation with product designers and asset owners are being planned.

For practitioners who are looking into integrating security into their CPS engineering processes according to the proposed method, important first steps are to develop a system understanding through creating function-based system diagrams as early as possible during system design, and to think about what drives (or is supposed to drive) their security-by-design decision making: risk, security goals, compliance, similar decisions made in earlier projects, functional requirements, or a combination or all? This is the groundwork for creating a useful security decision base and making more informed security decisions. Practitioners (CPS manufacturers or operators alike) who want to develop their own security libraries or support the author’s library-building are welcome to reach out to the authors.

The presented work also has implications for security policymakers. The problem analysis shows that for incorporating security by design into CPS engineering, decision makers need flexibility regarding the reasons and paths upon which they arrive at a security design decision. Security-by-design regulation should not prescribe specific security requirements, but instead require transparency and sound rationales for all security decisions made—and ideally also work toward a harmonized, machine-processable format to document these decisions.

## Figures and Tables

**Table 2:** Problems of existing methods.

Problem (and Causes)	Impact	Potential Solution (See Proposed Concept)
**1. “Undercover” security decisions:** System characteristics that do not include obvious security technologies but have an impact on the system’s security posture are not identified as security decisions. **Cause:** These decisions are not included in security checklists, because they do not look security-relevant at first sight but are “other domains’ business”.	Security decisions are missed during development and made without considering their security impact.	Do not identify security decisions based on potential security requirements (or the lack thereof), but based on security relevance of existing design decisions across all engineering domains.
**2. Lack of comprehensive guidance for security decision making:** There is no guidance that covers all decision-making paths (compliance-driven, risk-driven, goal-driven, library-supported). Especially, goal-driven and library-supported decision making is hardly present in state-of-the-art CPS security research. For risk-driven security decision making: fragmented methods for single steps in the decision-making-workflow, but none that give comprehensive guidance from problem understanding to decision making.	ICS/CPS engineers cannot turn to any single method they can use for their entire decision-making workflow. In addition, existing methods do not reflect the variety of reasons why they are making security decisions in reality. Both factors are lowering the engineers’ willingness to adopt cybersecurity methods during design.	Develop an overarching method that can incorporate different kinds of specialized methods, but that is simple enough to use by engineers that are not security experts.
**3. Shortcomings in system understanding:** There is little guidance and no comprehensive model for the system understanding phase at the beginning of risk-driven or goal-driven (and potentially library-supported) decision making.	Security decisions are made based on fragmentary system understanding. Security decisions and the assumptions that led to them are not documented systematically.	Develop a security decision-making concept with a systematic, guided system-understanding phase. Develop a comprehensive security system model that contains security-relevant information for all decision-making steps along all different decision-making paths (e.g., threats, risks, goals, requirements, measures). Make sure it is straight-forward to use system models when making security decisions.
**4. Lack of security decision traceability:** It is difficult to clearly understand and document the rationale for each security decision. **Cause:** This is a result from problems 2 and 3. If the system understanding is only documented fragmentarily, and the logical steps from the system understanding to making the security decisions are not interconnected in a comprehensive method and data model, it is difficult to trace back a security decision to why it was made—which decision-making path was followed, which goal was to be fulfilled, which risk was to be mitigated, which regulation was to be complied with, and which system functions were to be protected.	It is difficult to explain to third parties (e.g., clients or auditors), management, or even engineering colleagues why a security decision was made. When a system or its (threat) environment changes and security decisions need to be revised, they need to be made all over again because the rationales and underlying assumptions for the existing decisions are not known.	Develop an easy, efficient way to document the reason behind each security decision, regardless of decision-making path.
**5. Large amount of information to be digested:** The amount of information that needs to be processed by the security decision maker has always been large, and it is increasing. **Cause:** Many of the state-of-the-art approaches that improve guidance for security decision identification and making at the same time increase the amount of relevant information: Adding “undercover” security decisions increases the number of decisions to be made. Overcoming the mechanistic system view and adding human stakeholders, their goals or intentions, and relations and dependencies between system components makes the system model more complex. The value of library-supported approaches increases with library comprehensiveness, also increasing the amount of potentially relevant information. In addition, compared to software systems, the engineering of CPS (and especially ICS) involves more engineering domains than for a conventional software system, again increasing the amount of potentially relevant information for security decision making.	The overwhelming amount of relevant information makes it more difficult to identify the relevant pieces of information for a decision at hand. It also makes security decision making more complex and time-consuming.	Find efficient ways to filter and present the large amounts of security-relevant information to the security decision-maker.
**6. Lack of tool support:** There is no tool to support security decision making during system design. Lack of tool support was identified by many of the reviewed publications, especially those that work with libraries or ontologies or aim at automating certain parts of security decision making.	With the increasing comprehensiveness (and complexity) of decision making, it becomes increasingly unlikely that engineers can follow a methodology without the clear guidance of a tool. Attempting to consider all information without tool support is error-prone and time-consuming. Security decision making that is inefficient and time-consuming is very likely to be skipped altogether. If security decisions are made, documentation is not likely to be produced without a tool, and it probably cannot be exchanged digitally with other engineering domains.	Develop a tool that provides guidance through security decision making, efficiently stores and presents all required information, provides sound documentation with little overhead, and has interfaces to other engineering tools.

**Table 3:** Excerpts from function library categories and example function titles in each category.

Library Category	Exemplary Functions in This Category
IT administration	Manage clients; administrate users; …
Security services	Detect malware; monitor security events; …
Network operations	Synchronize time; resolve names; …
Automated control	Influence physical process; transmit measurements to control system; …
Operate and monitor	Display controller states; react to alarms; force controller outputs; …
Cloud	Offline data analysis; forecast operating data; …
Office operations	Save files; place an order; pay a bill; …
Engineering	Update PLC logic; test and debug PLC logic; calibrate sensor; …
Building automation	Monitor climate; operate lights automatically; …
Communication services	Transfer file to third party; transfer file internally; send e-mail; …
Process information services	Archive process data; analyze process data; …
…	…

**Table 4:** Security parameter attributes for example parameter “*logic update during operations*”.

ID and Title	SP091: Logic Update during Operations
Description	Some controllers have a configuration that allows the controller logic to be updated during operation (i.e., the controller does not have to be shut down).
Entities ^1^	PLC; Safety PLC
Possible values	Enabled; disabled
Attack indicators ^2^	Enabled
Security relevance	Updating during operation may be technically necessary. From a security point of view, however, it makes it easier to manipulate the controller, and because the PLC is in operation, the manipulated logic would then also be directly productive, regardless of system state.
Reference ^3^	/

^1^ Entities to which the security parameter may apply. ^2^ Parameter values that may be used in a security attack and thus regarded as critical. ^3^ Reference to related documents (guidelines, manuals, …). For some parameters or entities, more detailed descriptions of options are available, e.g., in vendor manuals.

**Table 5:** Use of the four diagram types in the security decision-making workflow.

Workflow Step	Activity	Diagram Type
1. Identify security decisions	Choose/modify functions	Function diagram
	Gain overview of security decisions that need to be made/have been made	Security decision diagram
2. Fill decision base	Provide network architecture context	Function diagram
	Define security goals	Security decision diagram
	Define High-Consequence Events	HCE diagram (opt.), Security decision diagram
	Model attack scenarios leading to HCEs	Attack diagram (opt.), Security decision diagram
	Define applicable regulation or standards	Security decision diagram
3. Make security decisions	Eliminate unnecessary functions	Function diagram
	Decide about security parameters and document rationale(s)	Security decision diagram

**Table 6:** Validation metrics to measure achievement of the validation goals (research questions), including results from the validation project described in this paper for the quantitative metrics. For the quantitative metrics, marked with an arrow (→), the results are explained in Section 4.4.

Validation Question	Metric
**Decision identification:** Do decision makers identify more security decisions?	**148** Decisions identified in **3.5 h**. **27** Additional ^1^ decisions identified
**Decision identification:** Do decision makers identify the same security decisions independently from each other?	**%** Decisions identified by all test persons **NOT MEASURED** (only one group of test persons)
**Decision making:** Can decision makers make security decisions autonomously based on the information offered?	**61** Decisions made in **4 h** (≈15 decisions/h) ^2^ **6** additional architectural decisions made **27 (44%)** Additional ^1^ decisions made **4 (7%)** Decisions changed ^1^ **0 (0%)** Decisions that could not be made → Were you the right person to make the decisions? → Why could you not make decisions/ which information was missing?
**Decision tracing:** Can a third party understand why each security decision was made this way?	**3** high-consequence events (HCEs), **10** attack scenarios, **11** security goals, **6** standards identified in **1 h**. **18 (30%)** Goal-driven decisions **22 (36%)** Risk-driven decisions **9 (15%)** Compliance-driven decisions **35 (57%)** Decisions based on a functional requirement ^3^ **0 (0%)** Decisions without a rationale → Likelihood of use for management/ client communication?
**Decision re-use:** Can artifacts used/produced during decision making be re-used in future projects?	**14** Applicable library functions (**13%** of library) **9 (64%)** library functions modified **0 (0%)** library functions newly created **135** Applicable security parameters (**86%** of library) → Likelihood of re-use in future projects?

^1^ Compared to the original project conducted without the new methodology. ^2^ 13 decisions not applicable to the project scope; 74 could not be made, due to time limitations. ^3^ or based on a functional restriction (some options not technically feasible).

## Data Availability

The data presented in this study are partially available upon request from the corresponding author. Some data are not publicly available, due to confidentiality agreements with an affected third party. These data are sensitive because they contain architectural details and security considerations that could potentially be used for a security attack.
